# The Accuracy of Finishing WEDM of Inconel 718 Turbine Disc Fir Tree Slots

**DOI:** 10.3390/ma14030562

**Published:** 2021-01-25

**Authors:** Jan Burek, Robert Babiarz, Jarosław Buk, Paweł Sułkowicz, Krzysztof Krupa

**Affiliations:** 1 Faculty of Mechanical Engineering and Aeronautics, Department of Manufacturing Techniques and Automation, Rzeszów University of Technology, 35-959 Rzeszów, Poland; jburek@prz.edu.pl (J.B.); robertb@prz.edu.pl (R.B.); sulkowicz@prz.edu.pl (P.S.); 2 Faculty of Mechanical Engineering and Aeronautics, Department of Materials Science, Rzeszów University of Technology, 35-959 Rzeszów, Poland; krupa@prz.edu.pl

**Keywords:** WEDM, finishing machining, Inconel 718, fir tree slot

## Abstract

Servicing aircraft engines sometimes requires manufacturing only a single piece of a given part. Manufacturing a turbine disc using traditional methods is uneconomical. It is necessary to use a different machining method recommended for small lot production. One of the proposed methods is WEDM (wire electrical discharge machining). The article presents the results of the research on finishing WEDM of Inconel 718 turbine disc fir tree slots. The influence of infeed, mean gap voltage, peak current, pulse off-time, and discharge energy on the shape accuracy, surface roughness, microcracks, and the white layer thickness were determined. Mathematical models were developed based on the DoE (Design of Experiment) analysis. The statistical significance of the models was verified with the ANOVA (Analysis of Variance) test. The machining parameters control methods that allow achieving the required shape accuracy, surface roughness, and surface layer condition were presented. The obtained surface roughness was Ra = 0.84 μm, the shape accuracy of the slot in the normal-to-feed direction was Δ*d* = 0.009 μm, the profile shape accuracy was Δ*r* = 0.033 μm, and the thickness of recast (white) layer was approximately 5 μm.

## 1. Introduction

Electrical discharge machining (EDM) is an alternative to traditional machining methods. The method consists of removing the material due to electrical discharges between the tool and the workpiece. As a result, EDM can be utilized in machining conductive materials regardless of their hardness [1,2]. EDM is widely used in manufacturing injection molds and in the automotive industry [3]. The continuous development of this machining method and innovations in the design of EDM machine tools allow applying the method in the medical sector and aerospace industry as well [4,5,6]. Wire electrical discharge machining (WEDM) is a type of electrical discharge machining in which the tool is an electrode in the form of a wire. The most commonly used is a brass wire of a diameter of 0.02 to 0.5 mm. The machining is conducted in a dielectric liquid and consists of removing the material as a result of melting and evaporation [7,8,9]. WEDM is a trepan type technology in which the erosion of the small amount of the machined material allows removing significant blank volume [10]. The occurrence of microcracks in the surface layer and the formation of a recast (white) layer [11] on a workpiece surface due to high temperature are notable shortcomings of the electrical discharge machining [12].

Considering the above, the method has not been commonly used in the manufacturing of critical machine parts, which include, i.e., an aircraft engine turbine rotor [13]. Depending on the accuracy and surface roughness, the machining of rotors may be only rough or can include a few finishing passes [14]. Finishing machining allows applying lower electrical parameters, which results in a lower discharge energy and reduced heat impact on the surface layer. Submerging the workpiece in the dielectric allows for effective cooling in the machining zone, which leads to stress in the surface layer. Lower discharge energy, and thus reduced heat impact on the surface layer, lead to lower stress in the layer, and as a result, to reduction in the microcracks [15,16]. Modern solutions in electrical discharge machining, as well as the materials with high hardness, allow avoiding microcracks in the surface layer and minimizing the thickness of the white layer on the surface of the workpiece. Mouralova et al. [17] investigated the influence of the Hardox 400 steel WEDM on the occurrence of surface defects. Gautier et al. [18] investigated the impact of pulse on time, pulse off-time, servo-reference voltage, and wire tension on the surface quality after WEDM of γ-TiAl. Deb et al. [19] investigated the influence of the peak current, pulse on time, and pulse off-time on the cutting speed, average surface roughness, total surface roughness, kerf width, and wire wear in Inconel 800 electrical discharge machining. Kulkarni et al. [20] investigated the machinability of NiTiNOL alloy in the WEDM process. Mouralova et al. [21] investigated the possibility of using WEDM for machining High-Entropy Alloys (HAE). Chaudhari et al. [22,23] investigated the surface quality of Shape Memory Alloys after WEDM. Kumar et al. [24] investigated the effect of pulse on time, pulse off-time, and wire tension on the surface roughness and the efficiency of the WEDM of Stellite alloy. Antar et al. [25] investigated the surface quality of Udimet 720 and Ti6246 alloy after WEDM with coated and uncoated electrodes. Thus, new possibilities in the machining of critical parts arise, especially for difficult-to-cut materials such as heat-resistant super alloys (HRSA), i.e., nickel superalloys [26]. Klocke et al. [27] presented the concept of using WEDM in manufacturing fir tree slots. Oniszczuk-Świercz et al. [28] investigated the effect of WEDM process parameters on the surface quality of Inconel 718 using coated brass wire. Sharma et al. [29,30] investigated the possibility of using WEDM in manufacturing Inconel 706 aircraft engine parts. Klocke et al. [31] compared the manufacturing of fir tree slots with the commonly used broaching method and the conceptual WEDM method. Newton et al. [32] assessed the influence of the WEDM roughing parameters on the recast (white) layer formation in the Inconel 718 machining. Aspinwall et al. [33] investigated the surface quality of Inconel 718 alloy after WEDM using the minimum damage generator technology. Due to the constant increase in aircraft engines performance, which is achieved while simultaneously reducing exhaust emission and noise (The Advisory Council for Aeronautics Research in Europe predicts a reduction in COx and NOx emission by 75% and noise reduction by 65% by 2050), newer materials are used, which are more and more difficult to machine [34,35]. The latest solutions in the design and control of WEDM machines allow achieving a surface roughness of 0.2 μm and a heat-affected zone (HAZ) thickness close to 0 μm [27]. In addition, the implementation of numerically controlled machine tools (CNC) allows programming any slot shape and does not require any special tools. On the other hand, one of the most significant disadvantages of the WEDM method is the long machining time, thus limiting its application mainly to single and small-lot production [34].

**Figure 1:**
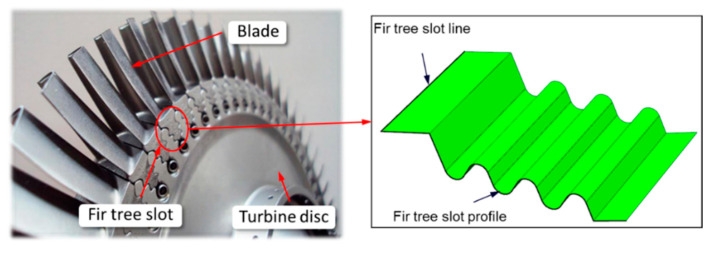
The surface of the fir tree slot [36].

In assembled turbine discs, the blades are attached to the disc with fir tree slots (Figure 1).

**Figure 2:**
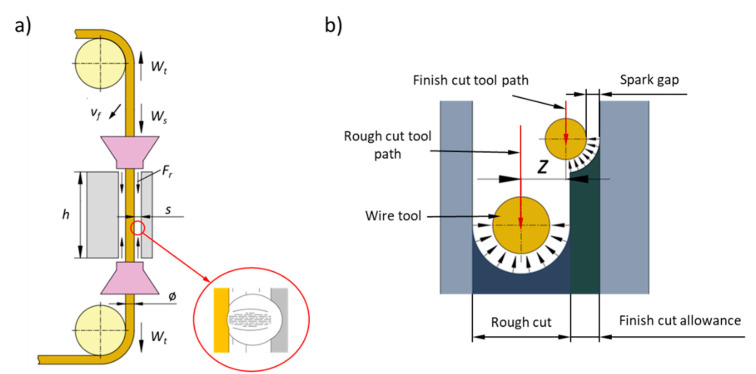
Hydromechanical and geometric parameter of wire electrical discharge machining (WEDM) process: (**a**) *W_s_*—wire running speed, *W_t_*—wire tension, *v_f_*—wire feed rate, *F_r_*—dielectric flow rate, *ø*—wire diameter, *h*—work piece’s height, *s*—spark gap, (**b**) *z*—infeed.

Currently, the most common method of machining the fir tree slots in turbine discs is broaching. Due to the complex shape of the tool as well as the difficulty in its design and manufacturing, the method is expensive and time-consuming. Therefore, alternative methods of machining the fir tree slots in the turbine discs are sought. Wire electrical discharge machining is one of the proposed methods. The method consists of a rough and finishing stage. High shape and dimensional accuracy and surface quality are achieved in finishing passes of an electrode [31]. The basic hydromechanical and geometric WEDM parameters are presented in Figure 2.

An important finishing machining parameter is the infeed of the electrode, which is the distance between the path of the electrode in roughing and finishing machining. The parameter significantly affects the shape accuracy, while the surface quality is largely influenced by the electrical parameters [37,38].

The research conducted so far has focused mainly on the roughing machining of Inconel 718 [8,39,40]. The studies of the finishing machining of Inconel 718 pertained to discharge energy, machining efficiency, and the effect of a single parameter [33,35,41]. The following article presents the results of the research on the influence of many parameters, such as electrode infeed, mean gap voltage, peak current, pulse off-time, and discharge energy on the surface quality and shape accuracy of Inconel 718 fir tree slots in turbine discs.

## 2. Materials and Methods

### 2.1. Experimental Material

Inconel 718 is a nickel-based alloy from the HRSA and HSTR (high-strength, thermal-resistant) superalloy group (Shanghai LANZHU super alloy Material Co., Ltd., Shanghai, China). Due to its properties, such as high strength, high temperature creep resistance, oxidation, and corrosion resistance, Inconel 718 has been used for parts that undergo high loads and high temperatures [42,43,44]. Table 1 presents the properties of Inconel 718 alloy.

Inconel parts make up approximately 50% of the mass of the aircraft engine, and it is being predicted that this value will increase. Inconel 718 is commonly used in manufacturing turbine discs, blades, combustion chambers, and gas turbines in the power industry [45]. The chemical composition of Inconel 718 is shown in Table 2.

### 2.2. Experimental Test and Measuring Stands

The test stand was built based on the Mitsubishi FA10S wire electrical discharge machine (Mitsubishi Electric Corporation, Tokyo, Kantō region, Japan). The machine tool was controlled by the Mitsubishi W31 Advance CNC control system (Mitsubishi Electric Corporation, Tokyo, Kantō region, Japan). The machine tool was powered by regenerative transistor pulse type using parameter notches, where a “notch” represents the unit for the actual value of a given parameter.

#### 2.2.1. Workpiece

**Figure 3:**
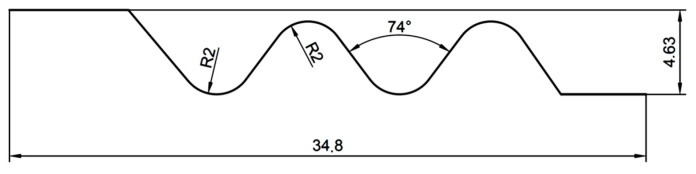
Tested fir tree slot profile.

The machined surface was the side of the fir tree slot, 30 mm wide. The dimensions of the slot profile are shown in Figure 3. The required surface roughness of the machined slots should be in the range of *Ra* = 0.8–1.25 µm, whereas the shape accuracy in the range of ± 5–25 µm.

#### 2.2.2. The Tool

For the experimental tests, a wire electrode with a diameter of 0.25 mm made of brass with a tensile strength of 900 N/mm^2^ was used. The adopted type of electrode is commonly used in the machine industry and is suitable for both rough and finishing machining.

#### 2.2.3. Test Conditions

Rough machining of the fir tree slot had been conducted in the same WEDM process, right before the finishing pass was made. The roughing parameters were selected for cutting steel according to the recommendations of the machine tool manufacture, modifying them according to the research carried out in the works [47,48]. Table 3 presents the constant machining parameters adopted in the experimental tests.

During the initial stage of the experimental tests, in order to determine the range of the *I_p_*, *U_m_* and *t_off_* parameter notches (for used *MP* power supply) for which the machining was possible, machining tests were performed. The values of the available parameter notches are presented in Table 4.

The minimum and maximum value of infeed were determined based on the previous research of the authors. The initial range of the infeed values calculated according to [49] was adapted in tests to find the minimum value allowing for the electric discharge between the wire electrode and the workpiece, and the maximum value allowing for the machining without short circuits or breaking the wire. Thus, the infeed range for finishing machining was *z* = 30–70 μm.

#### 2.2.4. Test Stand

**Figure 4:**
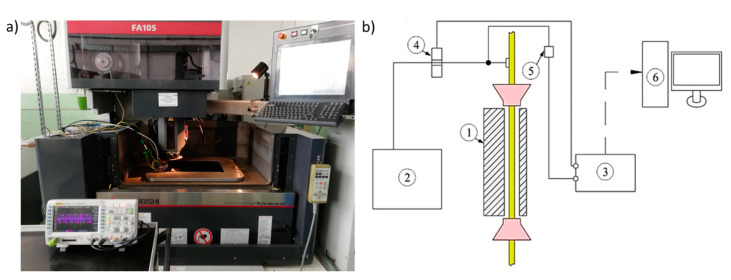
Test stand: (**a**) test stand view, (**b**) electrical parameters measurement scheme: 1—workpiece, 2—generator, 3—oscilloscope, 4—Pearson sensor, 5—voltage probe, 6—computer.

The test stand is presented in Figure 4. The voltage was measured with a Rigol RP1300H voltage probe (RIGOL Technologies, Beijing, China), and the current was measured with the use of a Pearson 2878 sensor (Pearson Electronics, Palo Alto, California, USA). The sensors were connected to a Rigol DS1074Z oscilloscope (RIGOL Technologies, Beijing, China). The measuring system allowed for recording voltage and current waveforms. The voltage was measured with the sensor with a measuring range of 400 V. The current was measured with a sensor based on the Hall phenomenon with the sensitivity of 0.1 V/A. The signals from the probe and sensor were recorded with the oscilloscope with a sampling frequency of 100 MHz and a recording time of 600 μs. The data were saved in flash memory and analyzed with the use of a computer.

**Figure 5:**
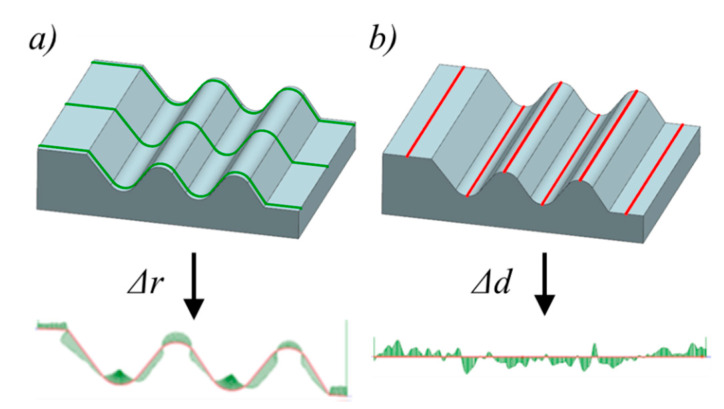
The measuring grid: (**a**) profile shape accuracy Δ*r*, (**b**) shape accuracy in the wire running direction Δ*d*.

The measurements of the shape accuracy of the fir tree slots were conducted with a Mahr XC20 conturograph (Mahr-Gruppe, Göttingen, Lower Saxony, Germany). The measurements of the profile accuracy Δ*r* were performed three times over the entire height of the sample: in the upper, middle, and lower sections (Figure 5a). The number of measuring points amounted to approximately 30,000. The measurements of the accuracy in the normal-to-profile (feed) direction Δ*d* were performed on the flat surface as well as on the internal and external radii (Figure 5b). The number of measuring points amounted to approximately 28,000.

**Figure 6:**
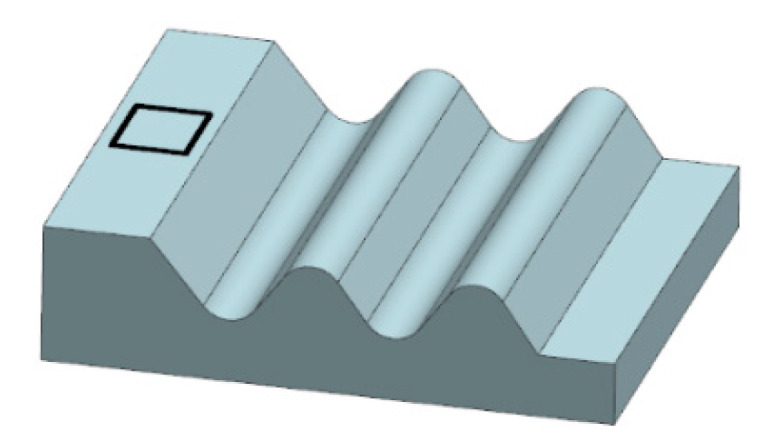
*Ra* roughness measurement area on the sample.

Surface roughness measurements were conducted on 1.4302 mm × 1.085 mm scan areas with a vertical resolution of 170.76 μm (Figure 6).

The area in the middle of the workpiece height was assumed due to the concentration of contaminants occurring in this zone during machining, and therefore, the worst machining conditions resulting in the highest roughness parameters [50]. The measurements were conducted with the use of an Infinite Focus G4 Alicona focus-variation microscope (Alicona Imaging GmbH, Raaba-Grambach, Styria, Austria).

### 2.3. Data Analysis Methods

The aim of the statistical analysis of the results was to obtain the regression models. The significance of the equation’s coefficients was determined with the Student’s *t*-test. The significance level was assumed at *α* = 0.05, and that value was used to test the null hypothesis *H*_0_. If the calculated value of the probability *p* was lower than the adopted *α* value, then the *H*_1_ hypothesis was assumed; otherwise, the null hypothesis *H*_0_ was assumed. To determine the goodness of fit of the models to the experimental data, the coefficients of determination *R*^2^ and ${\bar{R}}^{2}$ were calculated as follows:

(1) $$R^{2}\;=\;\frac{\sum_{i\;=\;1}^{n}{\left(\bar{y_{i}}-y_{i}\right)}^{2}}{\sum_{i\;=\;1}^{n}{\left(y_{i}-y_{i}^{\prime }\right)}^{2}}\;$$

where

- *y_i_*—measured value,
- $\bar{y}$_i_—theoretical value calculated from the model,
- $y_{t}^{\prime }$—arithmetic mean of measured values,
- *n*—number of measurements.

(2) $${\bar{R}}^{2}=\frac{\sum_{i=1}^{n}{\left(\bar{y_{i}}-y_{i}\right)}^{2}\left(n-1\right)}{\sum_{i=1}^{n}{\left(y_{i}-y_{i}^{\prime }\right)}^{2}\left(n-m\right)}\;$$

where

- *m*—number of interactions in a regression model.

The values of the coefficients of determination are in the 〈0,1〉 range, where the higher the value, the better the fit of the model to the experimental data.

The normality of the residual distribution was also tested using the Shapiro–Wilk test. For the determined probability (*p* > 0.05), the residuals are normally distributed. The statistical significance of the model was determined with the ANOVA test. All the statistical analyses were conducted using the JMP 12 software (SAS, Marlow, Buckinghamshire, United Kingdom) [51,52].

The statistical analysis of the experimental results was performed based on fitting the response surface models to the assumed input machining parameters. The works of authors [47] indicate the need to verify the influence of the infeed in the third power (*z^3^*). The response equation can be presented as a polynomial function in the general form:

(3) $$\begin{array}{ll} y\;=\;\beta_{0}\;+\;\beta_{1}I_{c}\; & +\;\beta_{2}U_{c}\;+\;\beta_{3}t_{off}\;+\;\beta_{4}z\;+\;\beta_{11}I_{c}^{2}\;+\;\beta_{22}U_{c}^{2}\;+\;\beta_{33}t_{off}^{2}\\ +\;\beta_{44}z^{2}\;+\;\beta_{12}I_{c}U_{c}\;+\;\beta_{13}I_{c}t_{off}\;+\;\beta_{14}I_{c}z\;+\;\beta_{23}U_{c}t_{off}\\ +\;\beta_{24}U_{c}z\;+\;\beta_{34}t_{off}z\;+\;\beta_{444}z^{3} \end{array}$$

where β—coefficients of the equation.

Complementing the research on the influence of electrical parameters, an analysis of the impact of the discharge energy *E* on the surface roughness and the condition of the surface layer was carried out, the discharge energy was calculated as follows:

(4) $$E\;=\;\int_{0}^{t_{on}}I_{p}\left(t\right)\cdot U_{m}\left(t\right)\;dt$$

where *t_on_*—pulse on time.

The statistical analysis was performed based on fitting the response surface models as well, whereas the response surface equation can be presented as a polynomial function in the general form:

(5) $$y\;=\;\beta_{0}\;+\;\beta_{1}E\;+\;\beta_{2}z\;+\;\beta_{11}E^{2}\;+\;\beta_{22}z^{2}\;+\;\beta_{12}Ez\;+\;\beta_{222}z^{3}$$

The determined ranges of parameters (Table 3) allow for developing the Design of Experiment (DoE). A four-parameter custom design was adopted with the use of a dedicated JMP 12 software. Table 5 presents the design of experiment.

## 3. Results

### 3.1. Test Results

The results of the measurement of the *Ra* surface roughness and profile shape accuracy Δ*r*, as well as the shape accuracy in the normal-to-feed direction Δ*d*, are presented in Table 6.

### 3.2. Surface Roughness

The quality of the machined surface depends mainly on the electrical parameters. The tests of the influence of electrical parameters were supplemented with the analysis of the impact of the discharge energy *E* on the surface roughness and surface layer condition. In order to determine the influence of the set electrical parameters on the surface roughness parameter *Ra*, a mathematical model was developed, based on the analysis of linear main effects, effects of two-way interaction, and a variable square effect in case of the infeed according to Equation (3).

The regression equation for the *Ra* surface roughness output can be presented as follows:

(6) $$Ra\;=\;1.546\;+\;0.67I_{p}\;+\;0.98I_{p}U_{m}\;+\;0.399I_{p}z\;+\;0.887U_{m}z\;+\;0.534z^{3}$$

**Figure 7:**
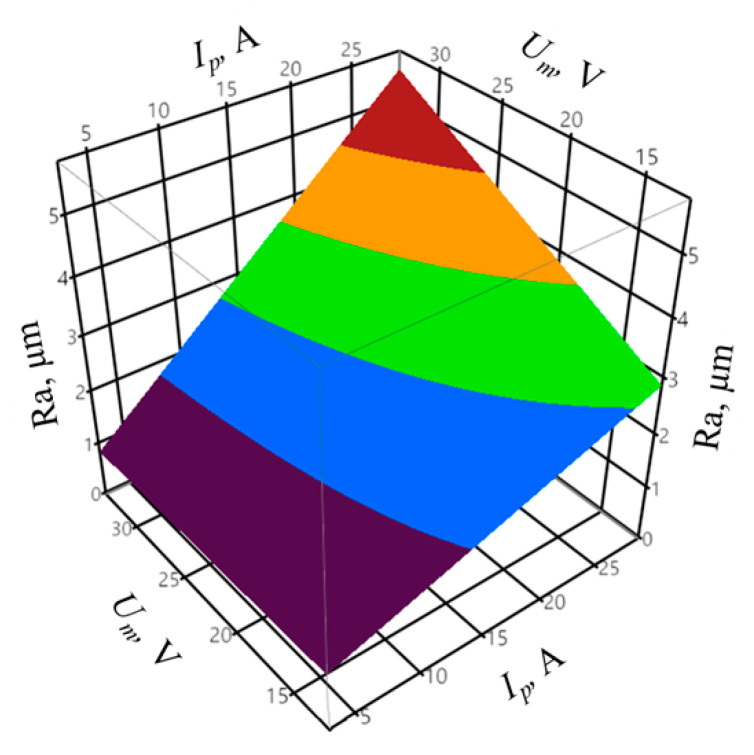
The relation between the surface roughness *Ra*, peak current *I_p_*, and mean gap voltage *U_m_*, with constant infeed *z* = 69 μm.

**Figure 8:**
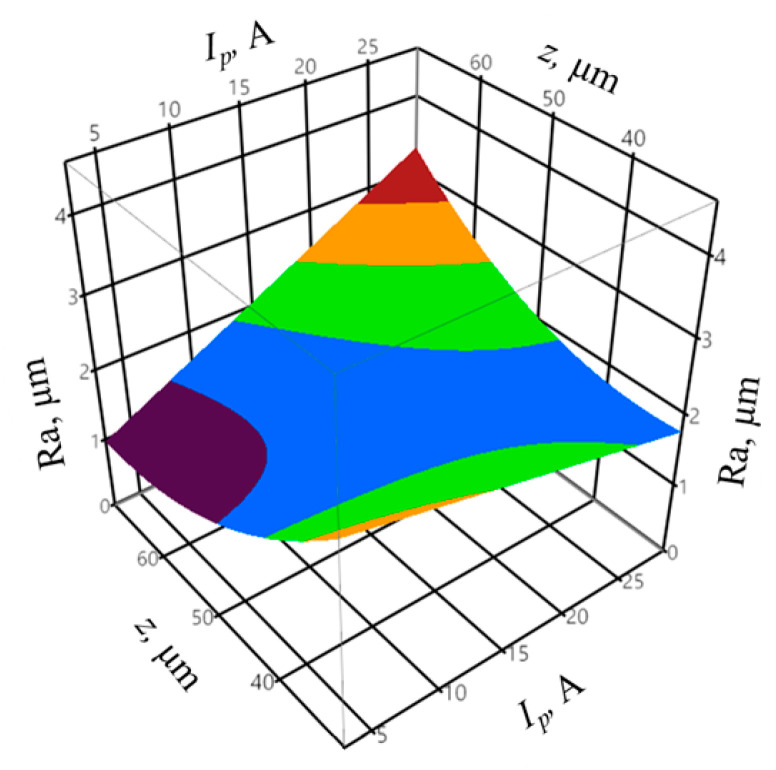
The relation between the surface roughness *Ra*, peak current *I_p_*, and infeed *z*, with constant mean gap voltage *U_m_* = 15.9 V.

In order to determine the goodness of fit of the model to the experimental data, the coefficient of determination *R^2^* and the adjusted coefficient of determination ${\bar{R}}^{2}$ were calculated according to Equations (1) and (2). The coefficient of determination was equal to *R^2^* = 0.96, whereas the adjusted coefficient of determination was equal to ${\bar{R}}^{2}$ = 0.95. The values of the coefficients indicate a very good fit of the model to the experimental data. The obtained probability value *p* = 0.4863 for the Shapiro–Wilk normality test suggests that the residuals were normally distributed. The verification of the statistical significance was conducted by one-way analysis of variance ANOVA. The probability *p* was equal to *p* = $1.677\;\times \;10^{-8}$, which confirms the statistical significance of the model expressed by Formula (6). The graphic interpretation of the developed model is presented in Figure 7 and Figure 8.

The peak current *I_p_* had the greatest impact on the surface roughness *Ra*. One can observe a significant interaction between the *I_p_* and *U_m_* parameters. The higher the *I_p_* value, the influence of the *U_m_* on the *Ra* surface roughness increases. In the case of the infeed, the value of approximately *z* = 40 µm resulted in the lowest surface roughness *Ra*. The pulse off-time *t_off_* does not significantly affect the value of the *Ra* surface roughness parameter. The measured surface roughness of *Ra* = 0.84–4.85 µm includes the roughness of *Ra* = 1.8–2.1 µm predicted by the machine tool manufacturer. However, it is possible to achieve a surface roughness *Ra* below 1 μm in the first finishing pass.

The analysis of the discharge energy *E* while varying the infeed *z* allowed obtaining, based on Equation (5), the regression equation for the output variable of the surface roughness *Ra* in the form:

(7) $$Ra\;=\;0.925\;+\;2.552E\;+\;0.853z^{2}\;+\;2.475Ez$$

**Figure 9:**
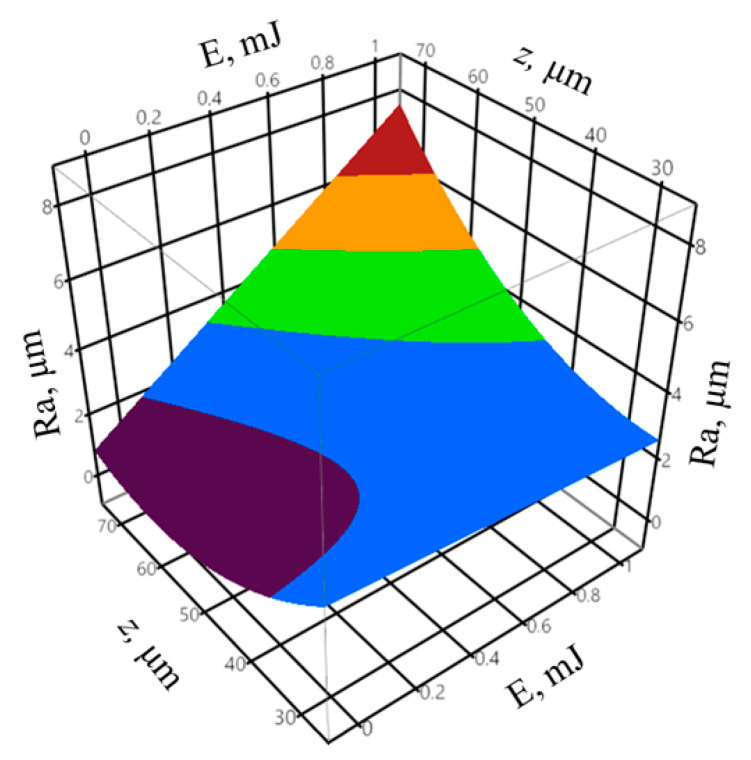
The relationship between the surface roughness *Ra*, discharge energy *E*, and infeed *z.*

The coefficient of determination was equal to *R*^2^ = 0.96, whereas the adjusted coefficient of determination was equal to ${\bar{R}}^{2}$ = 0.83. The values of the coefficients indicate a good fit of the model to the experimental data. The obtained probability value *p* = 0.5151 for the Shapiro–Wilk normality test allow concluding that the residuals were normally distributed. The verification of the statistical significance was performed by one-way analysis of variance ANOVA. The probability *p* was equal to *p* = $3.704\;\times \;10^{-6}$, which indicates the statistical significance of the model expressed by Equation (7). The graphic interpretation of the developed model is presented in Figure 9.

The high variation in the *I_p_* parameter, compared to mean gap voltage and pulse on time, has a significant impact on the discharge energy *E* value; thus, the influence of the energy on the surface roughness is similar to that of the peak current (Figure 7).

### 3.3. Surface Texture and Surface Layer

**Figure 10:**
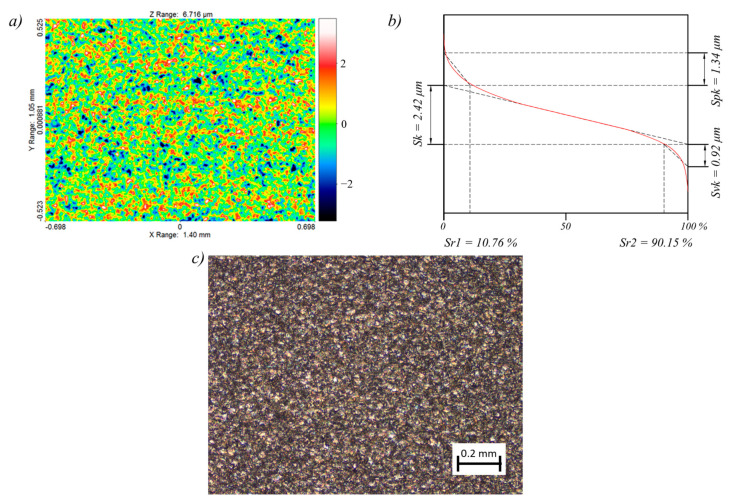
Surface topography and surface layer for sample 2: (**a**) contour plot, (**b**) Abbott–Firestone curve, (**c**) 10× magnification of the surface.

**Figure 11:**
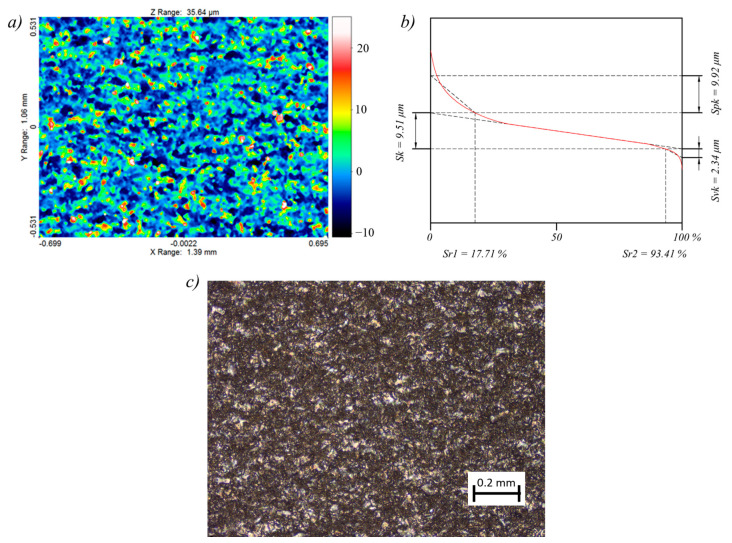
Surface topography and surface layer for sample 12: (**a**) contour plot, (**b**) Abbott–Firestone curve, (**c**) 10× magnification of the surface.

The results of the measurements of the surface texture and the surface layer for the test sample, for which the lowest (sample 2) and the highest (sample 12) surface roughness *Ra* were obtained, are presented in Figure 10 and Figure 11.

The surface topography of both samples was random and isotropic. The skewness for sample 2 was equal to *Ssk* = 9.25, and for sample 12, it was equal to *Ssk* = 1.39. The lower the value, the more rounded the peaks. A positive value indicates sharp peaks, which is disadvantageous in terms of surface interaction. The kurtosis for sample 2 was equal to *Sku* = 3.06, and for sample 12, it was equal to *Sku* = 5.91. The value close to 3 proves that the distribution of ordinates corresponds to the normal distribution, which in turn indicates an even distribution of peaks and valleys. In the case of the sample 12, significantly more valleys than peaks were registered. The root mean square roughness for sample 2 was equal to *Sq* = 0.98 µm, and for sample 12, it was equal to *Sq* = 5 µm. The maximum peak height and the maximum valley depth were equal respectively for sample 2 to *Sp* = 7.61 µm and *Sv* = 4.44 µm, and for sample 12 to *Sp* = 29.6 µm and *Sv* = 14.02 µm. The summit density for sample 2 was equal to *Sds* = 2798 pks/mm^2^, and for sample 12, it was equal to *Sds* = 1521 pks/mm^2^. The slight difference in the *St* and *Sz* parameters for both samples, in the range of 0.8–1.2 µm, indicates that not many random peaks and valleys occurred, which in turn proved that the machining was characterized by the stability of electrical discharges. Due to the surface interaction, the bearing area curves (Abbot–Firestone curves) were calculated as well for the machined sample 2 (Figure 10b) and for sample 12 (Figure 11b).

In order to assess the abrasion resistance, the reduced peak height parameter was adopted, which was equal respectively for sample 2 and 12 to *Spk* = 1.34 µm and to *Spk* = 9.92. Moreover, the lubrication fluid retention ability is described by the reduced valley depth parameter, which for samples 2 and 12 was equal respectively to *Svk* = 0.92 µm and *Svk* = 2.34 µm. The effective roughness depth is described by the core roughness height and was equal to *Sk* = 2.42 for sample 2 and to *Sk* = 9.51 for sample 12. The peak material component for sample 2 was equal to *Sr1* = 10.76%, and for sample 12 to *Sr1* = 17.71%, while the valley material component was equal to *Sr2* = 90.15 for sample 2 and to *Sr2* = 93.41 for sample 12.

**Figure 12:**
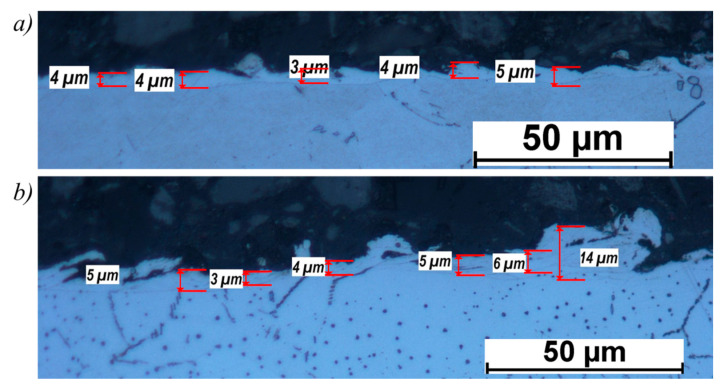
Surface layer: (**a**) sample 2, (**b**) sample 12.

A negative phenomenon occurring during electrical discharge machining is the formation of a white layer in the surface layer of a workpiece. Figure 12 presents the thickness of a white layer formed on the samples 2 (Figure 12a) and 12 (Figure 12b).

For sample 12, the white layer is irregular and is characterized by high thickness variation. On the other hand, for sample 2, the changes in height were minimal, in the range of ± 1 μm. Higher peak current *I_p_* contributed to significant local disturbances in the erosion of the material, which resulted in the deposition of an increased amount of the eroded material, which in turn led to an increase in the thickness of the white layer up to 12–14 µm.

**Figure 13:**
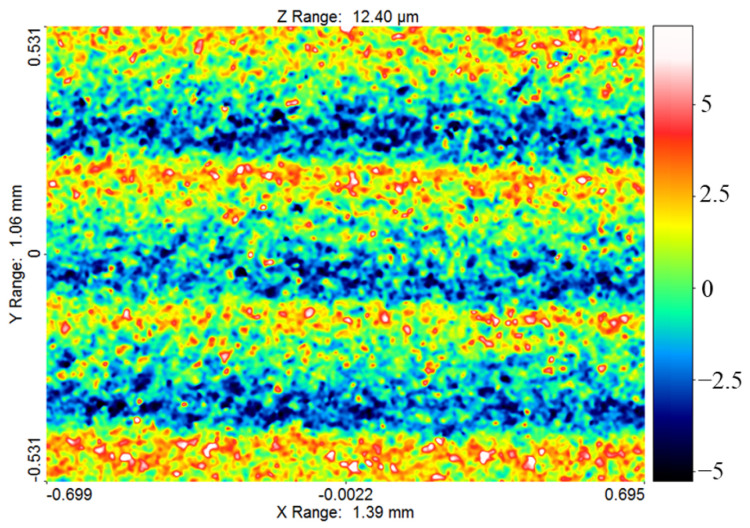
Surface topography for sample 9.

In addition, Figure 13 presents the topography of the sample 9 surface, which was the only one to bear the rarely occurring machining marks (visible in the direction of the wire running). The marks may be the result of regular electrode oscillations due to the selected electrical parameters and infeed.

### 3.4. Shape Accuracy

The regression equation for the output variable of the shape accuracy Δ*r*, based on Equation (3), can be presented as follows:

(8) $$∆r\;=\;\;-0.052\;-\;0.029I_{p}\;+\;0.006t_{off}\;-\;0.003I_{p}^{2}\;+\;0.001I_{p}t_{off}\;-\;0.00009t_{off}^{2}\;-\;0.0001U_{m}t_{off}\;\;-\;0.001I_{p}z\;+\;0.009U_{m}z\;+\;0.005z^{2}\;+\;0.005z^{3}$$

**Figure 14:**
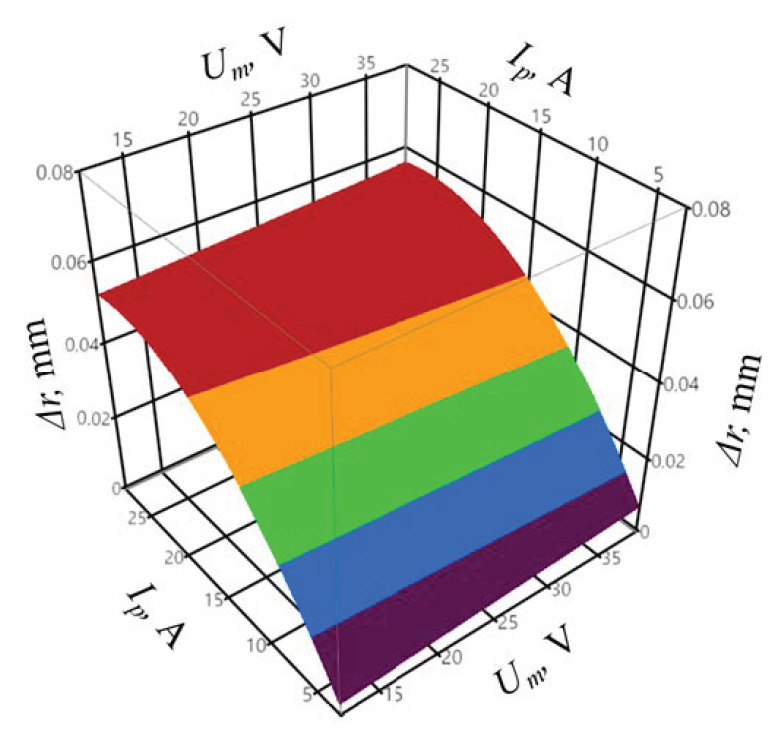
The relation between the shape accuracy Δ*r*, peak current *I_p_*, and mean gap voltage *U_m_*, with constant pulse off-time *t_off_* = 25 µs and infeed *z* = 70 µm.

**Figure 15:**
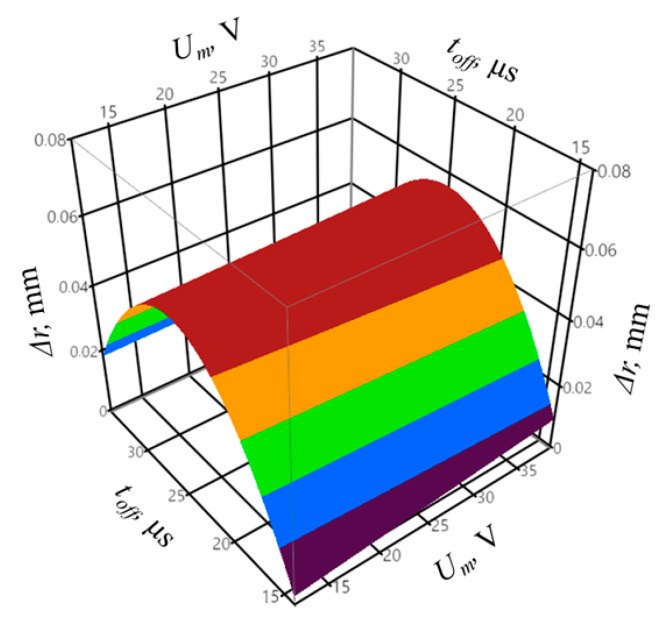
The relation between the shape accuracy Δ*r*, mean gap voltage *U_m_*, and pulse off-time *t_off_* with constant peak current *I_p_* = 28.6 A and infeed *z* = 70 µm.

**Figure 16:**
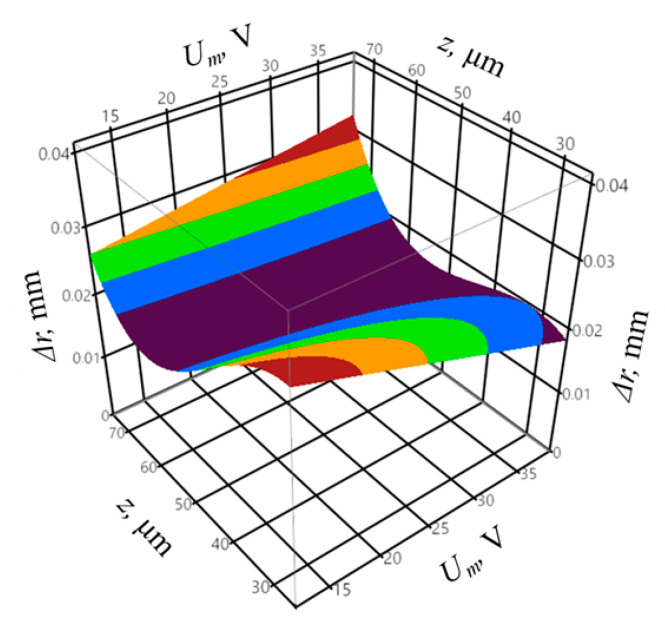
The relation between the shape accuracy Δ*r*, mean gap voltage *U_m_*, and infeed *z* with constant peak current *I_p_* = 28.6 A and pulse off-time *t_off_* = 17.2 µs.

The coefficient of determination was equal to *R*^2^ = 0.95, and the adjusted coefficient of determination was equal to ${\bar{R}}^{2}$ = 0.88. The values of the coefficients indicate a good fit of the model to the registered data. The obtained probability value *p* = 0.0631 for the Shapiro–Wilk normality test allows concluding that the residuals were normally distributed. The verification of the statistical significance was conducted by one-way analysis of variance ANOVA. The probability *p* was equal to *p* = 0.001153, which confirms the statistical significance of the model expressed by Equation (8). The graphic interpretation of the developed model is shown in Figure 14, Figure 15 and Figure 16.

**Figure 17:**
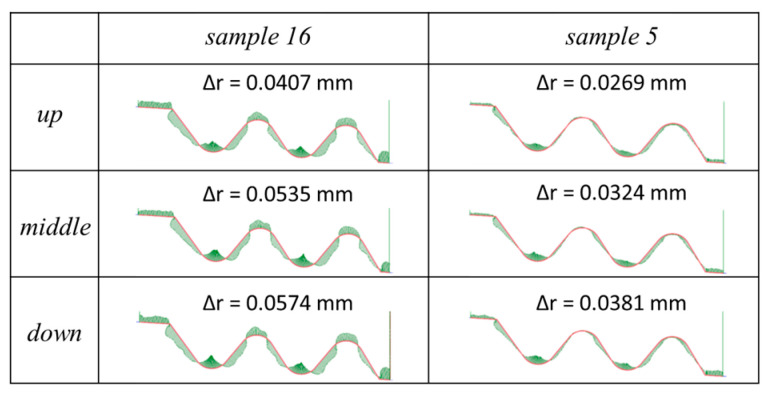
The distribution of the shape deviation of the profile Δ*r* for sample 16 (the highest deviation) and for sample 5 (the lowest deviation), scale ×60.

In the case of the infeed, the value for which the shape deviation of the profile Δ*r* was minimal proved to be approximately *z* = 50–60 µm. The highest profile deviation was achieved for the pulse off-time of approximately *t_off_* = 25 µs. The lowest value of the shape deviation of the profile was equal to Δ*r* = 32 µm. Figure 17 presents the distribution of the shape deviation of the profile Δ*r* at different heights of the workpiece for sample with the highest deviations (sample 16) and the lowest (sample 5).

A significant reduction in the deviations of the flat surfaces and outer radii has been achieved. In the area of internal radii, a slight decrease in the deviation values was noted.

The regression equation for the output variable of the shape accuracy in the normal-to-feed direction Δ*d*, based on Formula (3), can be expressed as follows:

(9) $$∆d\;=\;0.054\;+\;0.01I_{p}\;-\;0.002t_{off}\;+\;0.054U_{m}\;-\;0.0002I_{p}t_{off}\;+\;0.00002t_{off}\;+\;0.007I_{p}U_{m}\;\;-\;0.002U_{m}t_{off}\;+\;0.005U_{m}z\;-\;0.002z^{2}$$

**Figure 18:**
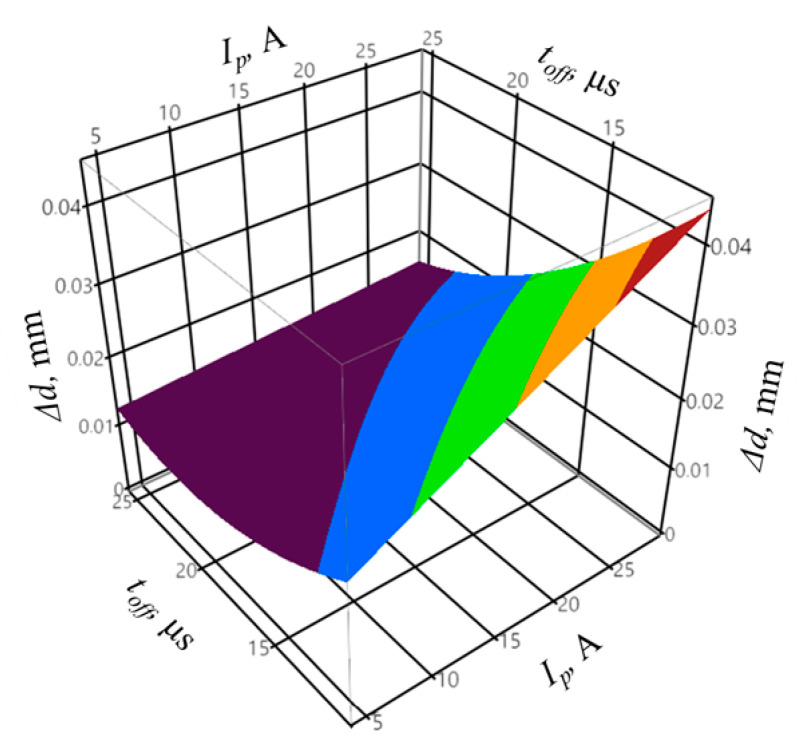
The relation between the shape accuracy in the normal-to-feed direction Δ*d*, peak current *I_p_*, and pulse off-time *t_off_* with constant mean gap voltage *U_m_* = 30 V and infeed *z* = 50.5 µm.

**Figure 19:**
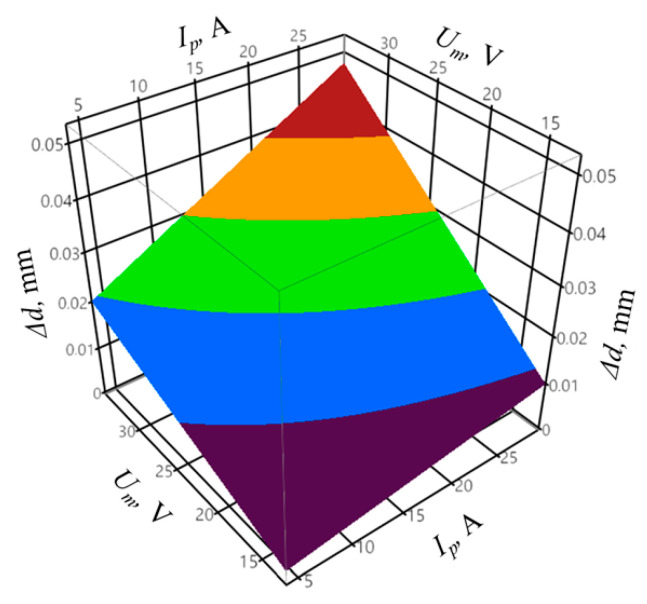
The relation between the shape accuracy in the normal-to-feed direction Δ*d*, peak current *I_p_*, and mean gap voltage *U_m_* with constant pulse off-time *t_off_* = 10.4 µs and infeed *z* = 70 µm.

**Figure 20:**
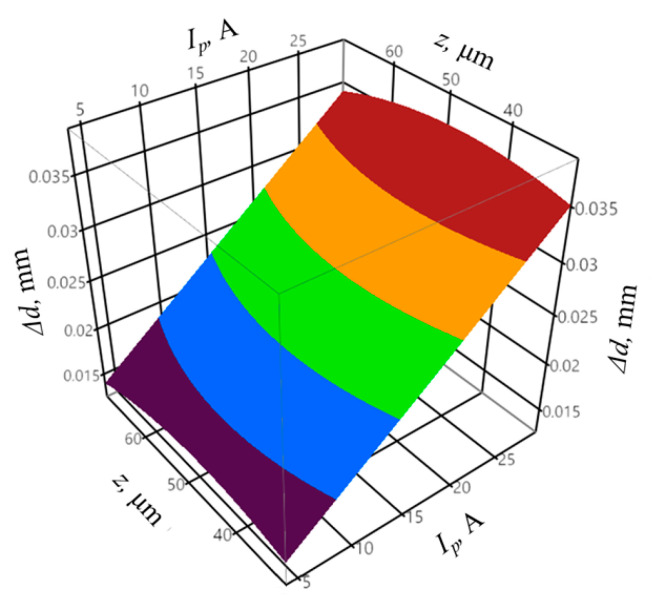
The relation between the shape accuracy in the normal-to-feed direction Δ*d*, peak current *I_p_*, and infeed *z* with constant mean gap voltage *U_m_* = 30 V and pulse off-time *t_off_* = 12.7 µs.

The coefficient of determination was equal to *R*^2^ = 0.95, whereas the adjusted coefficient of determination was equal to ${\bar{R}}^{2}$ = 0.90. The values of the coefficients indicate a very good fit of the model to the registered data. The obtained probability value *p* = 0.0809 for the Shapiro–Wilk normality test suggests that the residuals were normally distributed. The verification of the statistical significance was conducted by one-way analysis of variance ANOVA. The probability *p* was equal to *p* = 0.000252, which confirms the statistical significance of the model expressed by Equation (9). The graphic interpretation of the developed model is presented in Figure 18, Figure 19 and Figure 20.

**Figure 21:**
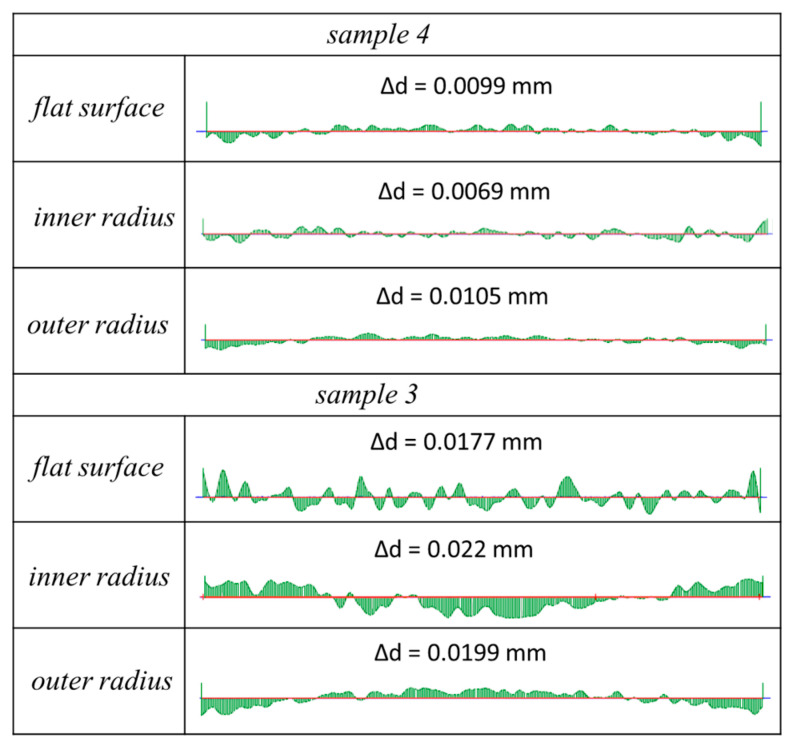
The distribution of the shape deviation in the normal-to-feed direction Δ*d* for sample 3 (the highest deviation) and for sample 4 (the lowest deviation), scale ×100.

One can observe a significant interaction between the *t_off_* and *U_m_* parameters. For the infeed of approximately *z* = 50 µm, the highest value of the deviation was recorded, contrary to the shape deviation of the profile Δ*r.* The lowest recorded value of the shape deviation in the normal-to-feed direction was equal to Δ*d* = 9 µm. Figure 21 presents the distribution of shape deviations Δ*d* on the flat surface as well as on the internal and external radii for sample with the highest (sample 3) and the lowest (sample 4) deviations.

One can observe that the distribution of the deviations corresponds with the characteristic deflection of the electrode. For sample 4, a significant reduction in the deviations was achieved, and the influence of the electrode’s deflection on the accuracy of the machining was minimized.

## 4. Conclusions

Due to economical reasons, manufacturing fir tree slots using traditional machining methods is justified only in large-scale or mass production. However, aircraft engine servicing requires the replacement of single parts, which makes traditional machining unprofitable. The conducted research indicates that the requirements of the industry pertaining to the surface roughness and shape accuracy in the normal-to-feed direction can be met, and the distribution of the deviations on the fir tree slot, except for the inside radii, can be significantly reduced.

The results of the conducted experimental tests allow formulating the following conclusions:

- A significant influence of peak current *I_p_* and mean gap voltage *U_m_*, and thus discharge energy *E,* on the surface roughness Ra was noted;
- Infeeds above approximately *z* = 50 μm have a considerable impact on the increase in Ra parameter;
- Pulse off-time *t_off_* did not have a notable influence on the surface roughness Ra;
- Higher peak current *I_p_* resulted in the significant increase in profile shape deviations Δ*r*;
- Δ*r* parameter significantly increased for the pulse off-time *t_off_* ≈ 20–30 μs, which can indicate higher electrode vibration amplitudę;
- The lowest Δ*r* parameter value was obtained for the infeed of z ≈ 40–60 μm and z ≈ 30 μm, and for the higher mean gap voltage *U_m_* (a significant interaction between *U_m_* and *z* parameters);
- The increase in *I_p_* and *U_m_* parameters leads to a notable increase in shape accuracy Δ*d*;
- A significant interaction between *I_p_* and *t_off_* parameters was noted, leading to the increase in Δ*d* deviations for low values of *t_off_* and high values of *I_p_*;
- The infeed slightly affected the deviation Δ*d*;
- Obtaining surface roughness in the Ra = 0.8–1.25 µm is possible even with only the one finishing pass;
- No microcracks were observed for any sample, the thickness of the white layer for sample 2 did not exceed 5 µm;
- The reduction in *I_p_* parameter from 23.5 A to 4 A resulted in the decrease in the thickness of the white layer of approximately 65%;
- A single finishing pass does not allow obtaining the profile shape accuracy Δ*r* within the tolerance of ± 5–25 µm;
- One can obtain the shape accuracy Δ*d* within the ± 5–25 µm in a single finishing pass;

The presented mathematical models can be the basis for the selection of electrical parameters and infeed in finishing WEDM for various slots geometry as well as for other Inconel 718 parts containing external and internal radii of approximately 20 to 40 mm.

Electrical discharge machining can meet some of the requirements in Ra surface roughness, surface layer, and shape accuracy Δ*d*. Thus, further research is needed, which should focus on the application of the successive finishing passes with the use of power supplies allowing for the machining with lower discharge energy, the main purpose of which should be to increase the profile shape accuracy Δ*r* and to lower the thickness of the white layer.

## Figures and Tables

**Table 1:** Properties of the annealed Inconel 718 alloy [32].

Property	Value
Density	8.19 g/cm^3^
Thermal conductivity	11.2 W/(m·K)
Electrical resistivity	127 μΩ·cm
Elastic modulus	200 GPa
Yield strength	150 ksi
Tensile strength	180 ksi
Tensile strength (1200 °F)	140 ksi
Hardness	89 HR_B_

**Table 2:** Chemical composition of Inconel 718 [46].

Alloy	Mass Percent (Mass%)													
	C	Si	Mn	Cr	Mo	Ni	Co	Ti	Al	Nb + T	P	S	Fe	Cu
Inconel 718	Max 0.08	Max 0.35	Max 0.35	17.0–21.0	2.8–3.3	50.0–55.0	0.04	0.65–1.15	0.2–0.8	4.75–5.5	Max 0.015	Max 0.015	18.5	Max 0.3

**Table 3:** Values of constant parameters adopted for the machining.

Technological Parameter	Value
Wire running speed *W_s_*, notch	12
Wire tension *W_t_*, N	19
Wire feedrate *v_f_*, mm/min	3.3
Dielectric flow rate *F_r_*, L/min	1.4

**Table 4:** Available notches for tested parameters.

Technological Parameter	Number of Notches
Peak current *I_p_*	13
Mean gap voltage *U_m_*	65
Pulse off-time *t_off_*	2

**Table 5:** The design of experiment according to notches and corresponding to them tested parameters.

Number of Sample	*I_c_*		*t_off_*		*U_m_*		*z*	*E*
	Notch	A	Notch	µs	Notch	*V*	µm	mJ
1	16	28.28	2	24.55	65	29.35	50	0.958
2	4	3.91	1	14.17	65	33.64	59	0.043
3	16	27.97	1	20.38	65	33.07	30	0.927
4	16	28.08	2	23.27	1	13.79	70	0.383
5	4	4.25	2	12.47	65	34.35	30	0.051
6	10	10.97	2	18.47	1	20.99	59	0.107
7	16	28.28	1	20.47	1	12.19	38	0.344
8	4	3.51	2	14.55	1	15.47	41	0.015
9	4	5.46	1	9.911	1	17.69	70	0.031
10	4	3.45	1	14.74	65	35.67	41	0.034
11	4	3.94	2	16.62	65	35.64	70	0.033
12	16	23.51	1	25.54	65	36.02	70	0.698
13	16	27.76	1	26.3	1	12.73	63	0.343
14	4	4.33	1	16.1	1	12.97	30	0.018
15	4	13.36	1	17.91	1	23.06	30	0.157
16	16	29.14	2	26.4	1	23.87	30	0.761
17	4	4.03	2	16.28	32	14.62	59	0.018
18	10	13.83	1	22.38	32	23.76	70	0.165

**Table 6:** The results of the measurement of the *Ra* surface roughness, profile shape accuracy Δ*r*, and the shape accuracy Δ*d*.

Number of Sample	*I_p_*	*t_off_*	*U_m_*	*z*		*E*	Ra	Δ*r*	Δ*d*
	A	µs	V	µm	mJ	µm	µm	µm	
1	28.28	24.55	29.35	50	0.958	3.304	0.042	0.015	
2	3.91	14.17	33.64	59	0.043	0.837	0.037	0.015	
3	27.97	20.38	33.07	30	0.927	2.91	0.04	0.02	
4	28.08	23.27	13.79	70	0.383	2.777	0.045	0.009	
5	4.25	12.47	34.35	30	0.051	1.776	0.033	0.016	
6	10.97	18.47	20.99	59	0.107	1.753	0.042	0.014	
7	28.28	20.47	12.19	38	0.344	1.571	0.044	0.013	
8	3.51	14.55	15.47	41	0.015	1.747	0.042	0.012	
9	5.46	9.911	17.69	70	0.031	1.604	0.037	0.009	
10	3.45	14.74	35.67	41	0.034	0.849	0.035	0.014	
11	3.94	16.62	35.64	70	0.033	0.897	0.048	0.011	
12	23.51	25.54	36.02	70	0.698	4.853	0.047	0.012	
13	27.76	26.3	12.73	63	0.343	2.574	0.045	0.014	
14	4.33	16.1	12.97	30	0.018	3.032	0.043	0.014	
15	13.36	17.91	23.06	30	0.157	2.524	0.046	0.013	
16	29.14	26.4	23.87	30	0.761	2.064	0.051	0.016	
17	4.03	16.28	14.62	59	0.018	0.923	0.036	0.01	
18	13.83	22.38	23.76	70	0.165	2.038	0.042	0.012	

## Data Availability

Data is contained within the article.
